# Acute Mesenteric Ischemia With Secondary Thromboembolism: A Rare Complication

**DOI:** 10.7759/cureus.9458

**Published:** 2020-07-29

**Authors:** Matthew R Figlewicz, Rachel E Bridwell, Josh Lowe, Amber Cibrario, Joshua Oliver

**Affiliations:** 1 Emergency Medicine, Brooke Army Medical Center, Fort Sam Houston, USA

**Keywords:** acute mesenteric ischemia, left atrial thrombus, secondary thrombus, vascular surgery, atrial fibrillation

## Abstract

Acute mesenteric ischemia presents a clinical challenge due to its subtle presentation and high mortality rate, which can mimic a variety of other conditions. Acute mesenteric ischemia requires a high index of suspicion, especially in those with comorbidities and risk factors such as hypertension, diabetes, atrial fibrillation, and peripheral arterial disease. The inciting thrombus commonly originates in the left atrial appendage or left atrium, embolizing to occlude mesenteric vessels, with resulting gut ischemia. Patients commonly present with post-prandial abdominal pain as mesenteric vascular demands increase, though diarrhea and gastrointestinal bleeding may be the presenting symptom. CT angiography of the abdomen and pelvis provides rapid confirmation of the diagnosis and visualization of the thrombus, aiding vascular surgical management. The authors present a novel case of a 69-year-old female with an acute mesenteric ischemia of her superior mesenteric artery and a second acute arterial thromboembolism to the right axillary artery visualized from her left atrial appendage.

## Introduction

With high mortality rates, acute mesenteric ischemia (AMI) is a challenging diagnosis, requiring a high index of suspicion. AMI occurs predominantly in the elderly or those with risk factors for thromboembolic disease, including hypertension (HTN), diabetes mellitus (DM), peripheral artery disease, and atrial fibrillation (AF). The authors present a case of AMI from thromboembolism, with a rare complication of second thromboembolism to the right axillary artery.

## Case presentation

**Figure 1:**
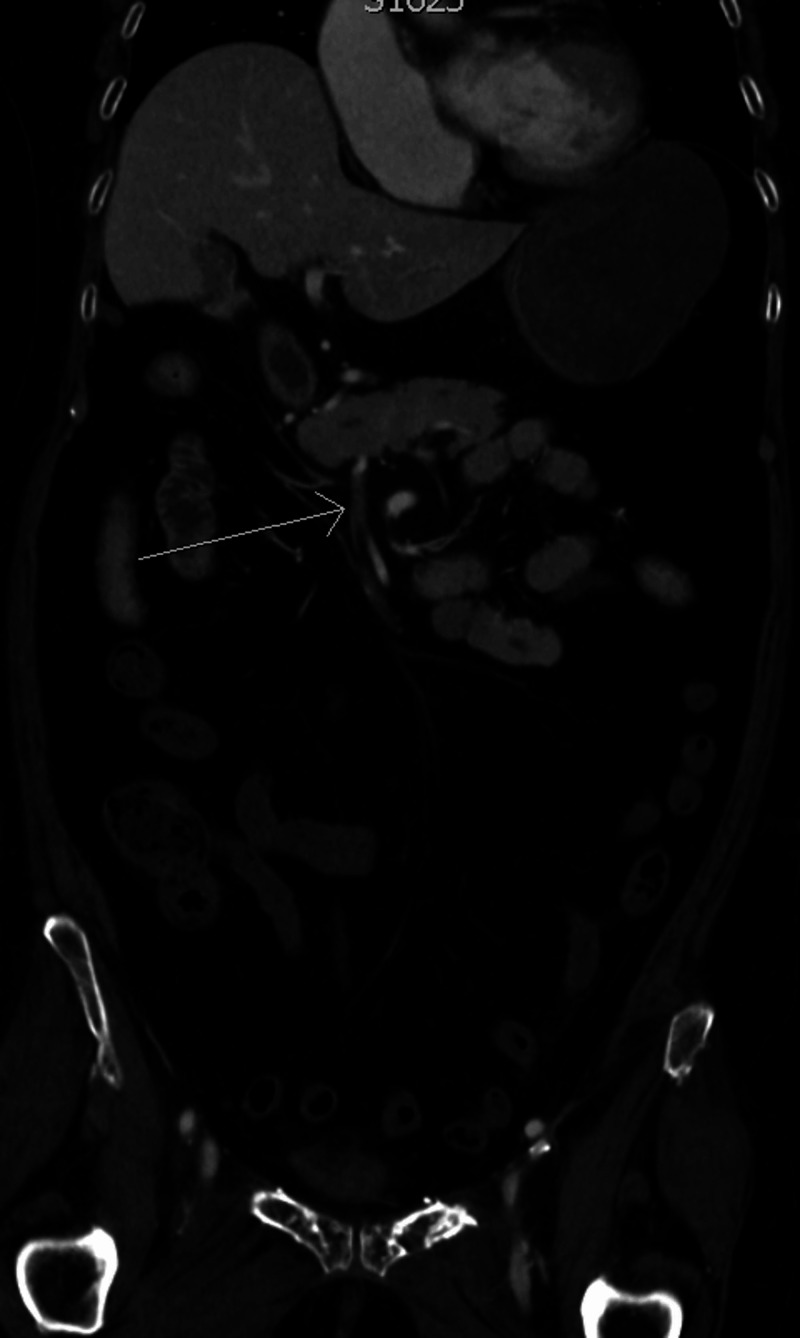
Coronal CT angiography demonstrating the superior mesenteric artery with complete occlusion with thromboembolism (arrow) and lack of distal flow

**Figure 2:**
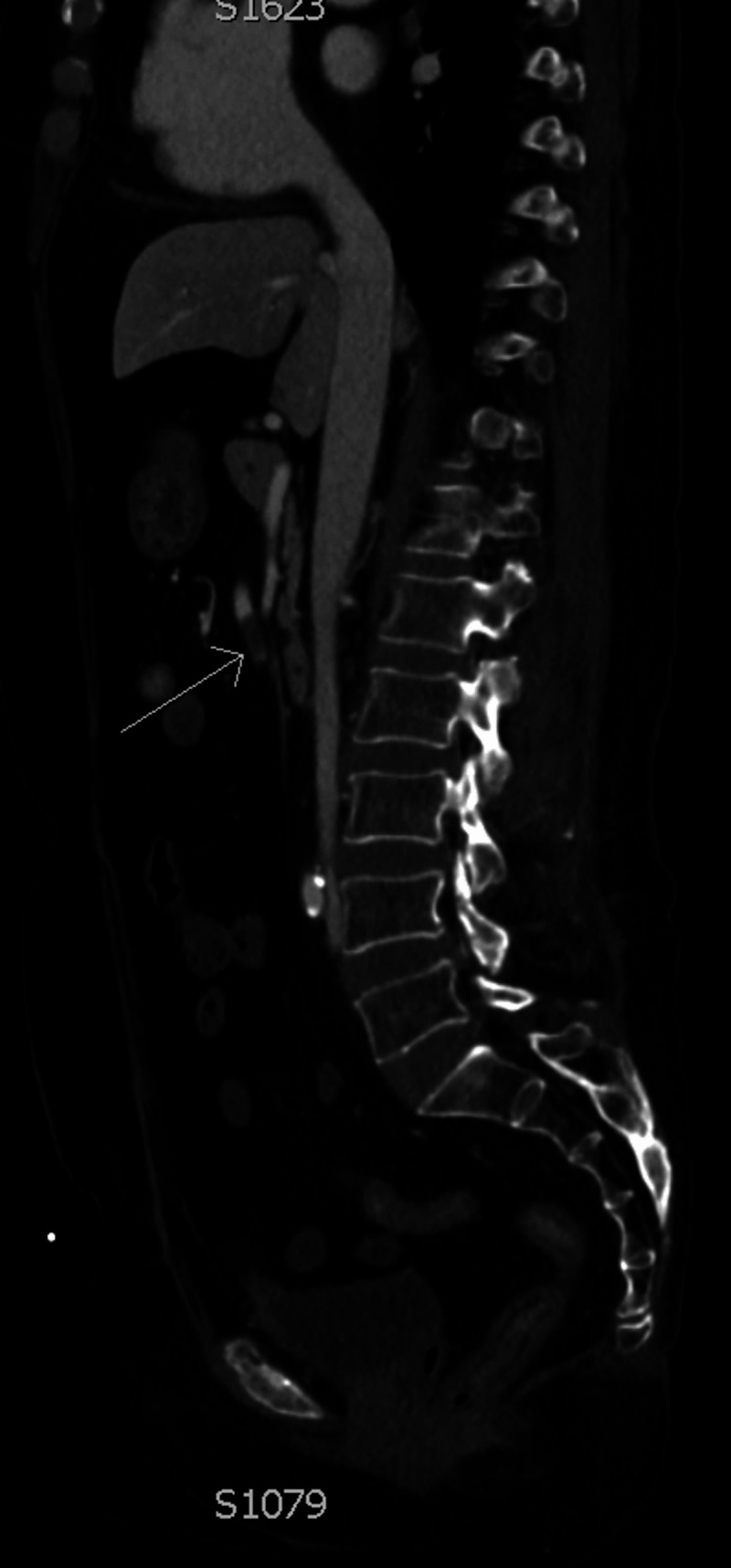
Sagittal CT angiography demonstrating the superior mesenteric artery with thromboembolism (arrow) and lack of distal flow

**Figure 3:**
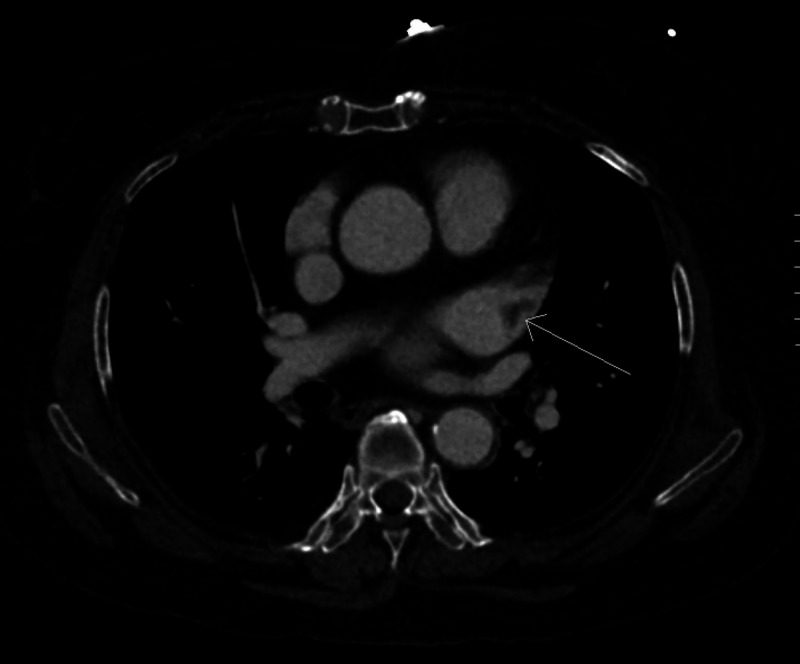
Coronal CT angiography demonstrating 1.8-cm left atrial appendage thrombus (arrow)

A 69-year-old female with a history of poorly controlled DM and AF was brought in by ambulance due to one hour of acute-onset mid-epigastric pain after eating. Under the instruction of her pain management specialist, she was on day 5 of a scheduled apixaban discontinuation in preparation of a lower back spinal stimulator scheduled the next day. On initial presentation, she was hypertensive with a blood pressure of 173/131 mm Hg and tachycardic with a heart rate of 104 beats per minute. On examination, the patient’s pain was not reproducible with abdominal palpation, with an ankle-brachial index greater than 1.0. Laboratory results were notable for serum lactate of 2.8 mmol/L (reference range: 0.5-2.2 mmol/L), leukocytosis of 19,700 white blood cells/microliter (reference range: 4,500-11,000 white blood cells/microliter), and creatinine of 1.13 mg/dL (reference range: 0.8-1.2 mg/dL). CT angiography demonstrated complete distal superior mesenteric artery (SMA) occlusion with associated ileal ischemia (Figures 1, 2) and a 1.8-cm thrombus in the left atrial appendage (LAA) (Figure 3). After consultation with vascular surgery service, the patient was started on a heparin drip and admitted to the surgical intensive care unit.

**Figure 4:**
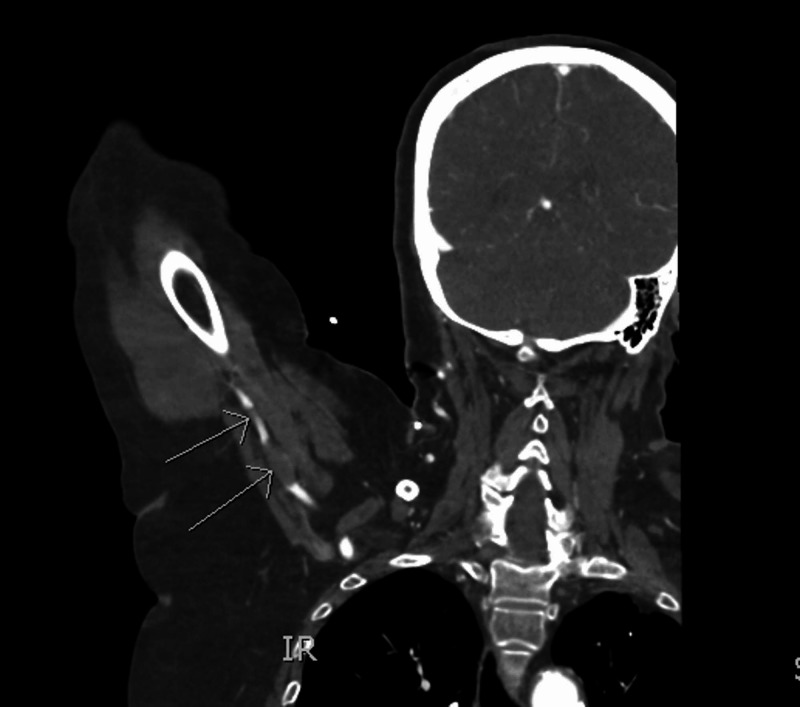
Coronal computed tomographyCT angiography of the right axillary artery demonstrating lack of contrast flow and thromboembolism (arrows)

On arrival, the patient complained that her right arm was cold and very painful. Repeat examination demonstrated marked discoloration, pain on passive wrist extension, and no pulses present on Doppler. A CT angiography of the right upper extremity showed an occlusive 2.5-cm thrombus in the axillary artery with mild reconstitution, though no flow distal to the elbow was noted (Figure 4). Additionally, interval resolution of LAA thrombus was highly suggestive of embolism to the right axillary artery.

Vascular surgery included a right upper extremity axillary-brachial-ulnar-radial embolectomy using an axillary approach with the return of palpable radial pulses. Despite therapeutic systemic heparin infusion, her clinical examination deteriorated, and an open SMA thrombectomy with removal of distal thrombus was performed with identification of a threatened small bowel. After a prolonged surgical ICU stay in which the patient required multiple abdominal surgeries, a tracheostomy, and refractory arrhythmias, she was discharged to an acute rehab facility without pain or sensory deficits to her right upper extremity.

## Discussion

Despite a challenging diagnosis, AMI is associated with AF, DM, HTN, and discontinuation of anticoagulation [1-4]. While abdominal pain occurs in anywhere between 60% and 100% of patients with AMI, gastrointestinal bleeding and diarrhea also occur in nearly half of patients, signifying a less common presentation [3,5,6]. Postprandial abdominal pain and food avoidance are also commonly reported. Rapid recognition and aggressive treatment of AMI are crucial for mitigating high mortality rates [1,2,7-9]. While D-dimer and lactate may support AMI, they cannot confirm the diagnosis, with a high sensitivity of 73-99% but low specificity of 47-55% [4,8].

Imaging provides critical information for diagnosis and management [6]. Bedside ultrasound with Doppler can evaluate the celiac and SMA branches, with sensitivity and specificity of 92-100% and 70-89% for arterial thrombus [5,8]. CT angiography, the gold standard, can be improved with a triple phase, allowing for the evaluation of plain, arterial, and venous phases [4,5,7,10,11]. Though thromboembolic lesions occur more commonly in proximal vessels, distal thromboemboli generate more extensive intestinal infarction [2].

Resuscitation of patients with AMI presents a challenging balance of optimizing hemodynamic stability without generating further ischemia. Since administration of vasopressors with alpha agonism can cause vasoconstriction and worsen ischemia, judicious crystalloid fluid administration should be used to maintain perfusion [12-14]. Early broad-spectrum antibiotics cover anaerobic translocation, improving outcomes [13,14]. If no contraindications, early initiation of systemic anticoagulation and consultation to a vascular surgeon are crucial as early revascularization demonstrates improved outcomes and decreased mortality [1,9,10]. Repeat abdominal surgical procedures allow for repeat assessment of the bowel, allowing for early identification of post-operative bowel necrosis, which has been shown to help prevent bowel resection and improve mortality [6,15,16].

Complications of AMI are common given the large insult to the mesenteric vascular supply. A potentially fatal complication, abdominal compartment syndrome, necessitates rapid surgical decompression with primary or repeat laparotomy [1,12]. AMI complicated by a second thromboembolism causing proximal acute arterial limb ischemia presents a previously unreported novel complication.

## Conclusions

AMI is a life-threatening disease, and it is important for emergency medicine providers to remember that risk factors for AMI can result in secondary thrombotic events. As demonstrated in this case, these additional thromboembolic events can occur outside of the mesentery, highlighting the importance of complete serial assessments for early identification of complications in these critically ill patients.
